# Social-Ecological Patterns of Soil Heavy Metals Based on a Self-Organizing Map (SOM): A Case Study in Beijing, China

**DOI:** 10.3390/ijerph110403618

**Published:** 2014-03-31

**Authors:** Binwu Wang, Hong Li, Danfeng Sun

**Affiliations:** 1 College of Resources and Environmental Sciences, China Agricultural University, No. 2 Yuan Ming Yuan west Road, Beijing 100193, China; E-Mail: binw_w@cau.edu.cn; 2 Key Laboratory of Agricultural Land Quality, Ministry of Land and Resources, No. 2 Yuan Ming Yuan west Road, Beijing 100193, China; 3 Institute of Agricultural Integrated Development, Beijing Academy of Agriculture and Forestry Sciences, No. 9 Shu Guang Hua Yuan Middle Road, Beijing 100097, China; E-Mail: lihsdf@sina.com

**Keywords:** social-ecological patterns, soil heavy metals management, geographical information system, self-organizing map, Beijing

## Abstract

The regional management of trace elements in soils requires understanding the interaction between the natural system and human socio-economic activities. In this study, a social-ecological patterns of heavy metals (SEPHM) approach was proposed to identify the heavy metal concentration patterns and processes in different ecoregions of Beijing (China) based on a self-organizing map (SOM). Potential ecological risk index (RI) values of Cr, Ni, Zn, Hg, Cu, As, Cd and Pb were calculated for 1,018 surface soil samples. These data were averaged in accordance with 253 communities and/or towns, and compared with demographic, agriculture structure, geomorphology, climate, land use/cover, and soil-forming parent material to discover the SEPHM. Multivariate statistical techniques were further applied to interpret the control factors of each SEPHM. SOM application clustered the 253 towns into nine groups on the map size of 12 × 7 plane (quantization error 1.809; topographic error, 0.0079). The distribution characteristics and Spearman rank correlation coefficients of RIs were strongly associated with the population density, vegetation index, industrial and mining land percent and road density. The RIs were relatively high in which towns in a highly urbanized area with large human population density exist, while low RIs occurred in mountainous and high vegetation cover areas. The resulting dataset identifies the SEPHM of Beijing and links the apparent results of RIs to driving factors, thus serving as an excellent data source to inform policy makers for legislative and land management actions.

## 1. Introduction

Accumulation of heavy metals in agricultural soils may cause serious problems to human well-being by influencing soil quality, groundwater and food chains [1,2,3,4,5]. For the last two decades, instead of natural factors, anthropogenic activity has significantly increased the circulation of toxic metals through soil, water and air. In China, a growing public concern has been focused on the trace elements environment owing to the rapid industrialization, urbanization and increasing reliance on agrochemicals in the last two decades [6,7,8]. Thus the complex system management of trace elements requires understanding the interaction between the natural system and human socio-economic activities (social-ecological patterns), not only to monitor the distribution status, but also to identify the patterns and processes in different ecoregions or ecosystems. Each ecoregion may respond relatively homogeneously to human activity or management actions [9,10,11,12,13,14]. 

Identifying and quantifying the social-ecological patterns of heavy metals (SEPHM) is a challenging task to due to the variety and complexity of social-ecological data [7,10,15,16,17,18]. Conventional multivariate methods are somewhat limited for interpreting the non-linear and complex dynamic nature. Agent models from biologically inspired machine intelligence have been proposed recently for analyzing and processing complex data to understand the ecological and physiological functioning of life systems [12,19,20], including artificial neural networks, genetic algorithms, support vector machines, individual-based models, cellular automata, fuzzy models, *etc*. [12,21,22] . Extensive information and examples can be found in Recknagel [23].

SOM is a very interesting and promising classification approach employing an innovative and data-driven classification method based on unsupervised artificial neural networks. Its capabilities of clustering, classification, estimation, and prediction have been used in a widely spread range of disciplines, including engineering, agriculture, health, environment management, and remote sensing image classification, *etc*. The SOM component planes can reveal very useful information to interpret results that remain hidden with the traditional approaches, such as the principal component analysis and hierarchical cluster analysis [24,25,26]. 

The heavy metal contamination of Beijing soils has been widely reported. Huo *et al.* [4] assessed the spatial variability of heavy metals with a total of 1,018 samples covering the entire Beijing agricultural soils area using Geostatistics, furthermore, combining Geostatistics with Moran’s I analysis to produce high quality heavy metals interpolation maps [27,28]. Jiang *et al.* [5] and Wang *et al.* [29] assessed the potential eco-risk of heavy metals in agricultural and urban soils, respectively. Many investigations have been done on the heavy metal pollution in different land uses of Beijing [29,30,31,32,33,34,35]. Li *et al.* [36] attempted to quantify the spatial linkages of the heavy metals in Beijing agricultural soil using complex network theory in order to identify their diffusion evolutionary mechanisms. However, there is still a notable lack of the social-ecological patterns study to elucidate the underlying processes between the natural system and human socio-economic activities with heavy metals and the remediation methods and policies. 

Therefore, the objectives of this study were to explore the potential of the SOM approach to identify the SEPHM in Beijing, and to propose individualized approaches to the management of the soil heavy metal pollution.

## 2. Study Sites

**Figure 1:**
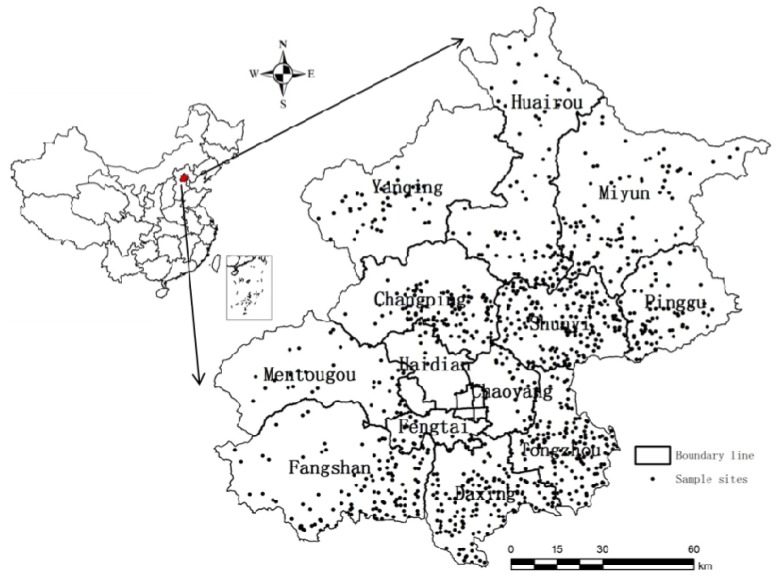
Distribution of sample sites in the study area.

Beijing, with an estimated area of 16.4 thousand km^2^, is located in the northwestern part of China’s north plain, generally between longitude 115°24'–117°30'E and latitude 39°38'–41°05'N. Its elevation slopes downward from 2,250 m in the northwest to 10 m in the southeast, and the mountainous area covers about 62% and plain 38% of the whole area (Figure 1). 

The area has a temperate continental monsoonal climate with an annual average temperature of 11.8° (average maximum 26° in July and average minimum −5° in January). The annual average temperature difference is 30.4°, while the daily average temperature difference is 11.4°. Annual precipitation in this area is 470–660 mm, about 60% of which comes in July and August. Annual average evaporation is 1,800–2,000 mm. The area is the source of five big rivers, the Yongding, Chaobai, Beiyun, Jiyun and Daqing. Annual average runoff is about 1.8 × 10^9^ m^3^, but had decreased to 1.3 × 10^9^ m^3^ by the end of the last century. The main soil types include drab soil, brown soil and skeleton soil in mountainous areas, and fluvo-aquic soil in plain areas. The population of the study areas was about 20.69 million, and the vehicle population reached 5.4 million in 2012. Heavy metals management is an important and complicated factor in the development of an ecological environment strategy in Beijing, because its environmental problems might represent the future of the other metropolis in China [37].

## 3. Material and Methods

### 3.1. Sampling and Sample Processing

In this study, 1,018 soil samples were collected in Beijing in 2006 using an irregular stratified sampling technique based on the agricultural land distribution and land use type maps [4]. All the samples' geographical locations were recorded in the WGS84 geographical system in order to process the data into the Geographical Information System (GIS). More details of the soil sampling procedure can be found in the guidelines described in the monitoring protocol [4,5]. The metal concentrations were determined by the methods described in the Chinese Environmental Quality Standard for Soils [38]. The Cr, Ni, Cu, and Zn concentrations were analyzed by flame atomic absorption spectrophotometry after digestion in a mixture of HCl, HNO_3_, and HClO_4_. Pb and Cd were analyzed by graphite furnace atomic absorption spectrophotometry, and the As concentration was determined by potassium borohydride silver nitrate spectrophotometry. In addition, the Hg concentration was determined by cold atomic absorption spectrophotometry after digestion with a mixture of H_2_SO_4_, HNO_3_, and KMnO_4_. Quality assurance and quality control procedures were conducted using the standard reference material Geochemical Standard Soil.

### 3.2. Social-ecological Data

In China, communities and/or towns are the smallest administrative units and usually act as the basic unit for planning and management purposes [39]. Environmental problems have been especially significant for the development of Beijing as the capital of China. The classification of heavy metals social-ecological patterns at the town-level will provide useful information for the establishing sustainable environmental management strategies in Beijing. Therefore, the town-level scale was the basic unit in this study.

**Figure 2:**
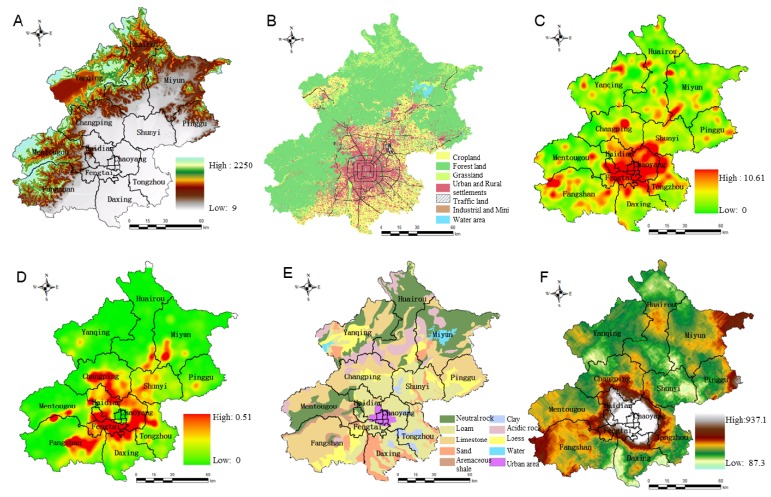
Main spatial data on the map of Beijing. (**A**) Elevation; (**B**) Actual land-use map; (**C**) Road Density; (**D**) Industrial and Mining Land Density; (**E**) Map of soil-forming parent material; (**F**) Map of potential ecological risk index (RI).

Numerous factors need to be considered for ecoregional characteristics clusters. It is a fundamental point to understand the correlations between ecological factors and heavy metals concentration. According to the previous literature investigations, the major inputs of trace elements to agricultural soils including atmospheric deposition, livestock manures, fertilizers and agrochemicals, sewage irrigation, sewage sludge, and some other sources [7,40]. Table 1 lists the contribution and the rank of each source to each individual heavy metals in soils. The complex sources of heavy metals are quantified and our classification datasets are also based on previous works on the spatial autoregression model [41] and risk grade assessment for heavy metals concentration of Beijing [5,27,42]. Additional geodata are from the Statistics yearbook, field surveys and spatial databases (Table 2, Figure 2). 

Town-wise statistics metadata mainly consist of these parameters: human population density of each town (ind.·km^−2^), livestock (unit·km^−2^), fertilizers and agrochemicals input (t·km^−2^), land use cover data (km·km^−2^ or percent distribution), elevation (m), precipitation (mm). Among them, land use cover data has six categories including Normalized Difference Vegetation Index (NDVI), industrial and mining land (percent distribution), river (km·km^−2^), road (km·km^−2^), single cropped and double cropped land (percent distribution). We gathered detailed information on bovine, ovine, porcine and avian livestock at the town-wise level for the year 2007. Livestock unit (LSU) was calculated in a standardized manner [43]. Overall, they correspond to a total of 60,657 livestock units in the study area. Human population data and fertilizers and agrochemicals input data were obtained from the Statistics yearbook of Beijing. Precipitation data was supported by China Meteorological Data Sharing Service System [44], and the monthly average of precipitation from 2000 to 2010 were calculated. 

**Table 1:** The contribution and the rank of sources to heavy metals in soils.

Sources	Determined Elements								Reference
	Cr	Ni	Zn	Hg	Cu	As	Cd	Pb	
Atmospheric deposition	✓✓✓	✓✓✓	✓✓	✓✓✓	✓✓	✓✓✓	✓✓	✓✓✓	[7]
Livestock manures	✓✓	✓✓	✓✓✓	✓	✓✓✓	✓✓	✓✓✓	✓✓	
Irrigation water	✓	✓✓	✓✓	✓	✓	✓	✓	✓	
Sewage sludge	✓	✓	✓	✓	✓	-	-	✓	
Fertilizers	✓✓	✓✓	✓✓	✓✓	✓✓	✓✓	✓✓	✓✓	
Agrochemicals	-	-	✓	-	✓✓	✓	-	-	
Industrial plants	✓✓	✓	✓✓✓	✓✓	✓✓	✓	✓✓	✓✓✓	[27,41]
Soil parent materials	✓✓✓	✓✓✓	✓✓	✓✓	✓✓	✓✓	✓	✓	
Mining	✓	✓	✓✓	✓✓	✓✓✓	✓	✓✓	✓	

**Table 2:** Metadata used to classify the social-ecological patterns of heavy metals.

Group	Variable	Abbreviation	Unit	Last Update
Demographic	Population Density	PD	ind.·km^−2^	2007
Agriculture structure	Livestock Units	LSU	unit·km^−2^	2007
	Fertilizers and Agrochemicals Input	FAI	t·km^−2^	2007
	Double Cropping	DC	%	2007
	Single Cropping	SC	%	2007
Geomorphology	Digital Elevation Model	DEM	m	2000
Climate	Precipitation	PREC	mm	2000–2010
Land use cover	Industrial and Mining Land	IML	%	2008
	Normalized Difference Vegetation Index	NDVI	-	2008
	River Density	RID	km·km^−2^	2008
	Road Density	ROD	km·km^−2^	2008
Soil-forming parent material	Loess	LOE	%	2006
	Loam	LOA	%	2006
	Sandstone	SAS	%	2006
	Arenaceous shale	ARS	%	2006
	Limestone	LS	%	2006
	Acidic rock	AR	%	2006
	Clay	CL	%	2006
	Neutral rock	NR	%	2006
Potential Ecological Risk index	Risk Indices	RI	-	2006

Land use cover data obtained from Beijing Municipal Bureau of Land and Resources, while the cropping patterns were classified by the Moderate Resolution Imaging Spectroradiometer (MODIS) image [45]. All the related data were stored and managed in the Beijing agricultural resources and economic data system [46]. The eco-toxicity of heavy metals depends to a great degree on their bioavailability in soils and their toxicological factors and soil properties (e.g., soil organic matter, soil pH, mineral contents), when estimating their bio-availability to animals or human health [29]. As for the purpose of land use management, the procedures based on the total heavy metal contents in soils were employed in this study. A number of methods have been suggested to quantify the enrichment of heavy metals in contaminated soils, such as Contamination factor (CF), Enrichment factor (EF), Nemerow index (NI)，Health risk index (HRI), Potential ecological risk index (RI) [47,48,49]. Among them, CF and RI are the typical representative pollution indexes, which have a wide range of application [5]. RI, also called the Hakanson potential ecological risk index, integrates the “toxic-response” factor and pollutant concentration of a given pollutant. *E_r_^i^* reflects the potential health risk in an ecosystem to a certain degree. The quantitative equation of the RI of a given pollutant was defined as follows:

*E_r_^i^ = T_r_^i^ × C_r_^i^*

where *T_r_^i^* is the toxic-response factor for a given pollutant, and *C_r_^i^* is the contamination factor. *C_r_^i^* was calculated by the measured concentration of metal *i* in the sample divided by the reference value. In this study, environmental quality standard secondary grade for soils, a soil limitation to ensure agricultural production and human health was applied [38]. The *T_r_^i^* values of metals were as follows: Hg (40) > Cd (30) > As (10) > Cu (5) = Pb (5) = Ni (5) > Cr (2) > Zn (1) [5]. The following terminologies are used to describe risk levels: *E_r_^i^* < 40, low potential ecological risk; 40 ≤ *E_r_^i^* < 80, moderate potential ecological risk; 80 ≤ *E_r_^i^* < 160, considerable potential ecological risk; 160 ≤ *E_r_^i^* < 320, high potential ecological risk; and *E_r_^i^* > 320, very high ecological risk. To facilitate clustering 253 towns by SOM, the biophysical dataset (such as CF and RI of each elements) was interpolated into a grid by Kriging, and the average of these indices were resampled at a town-wise level to match the socio-economic data for the further utilization of SOM.

### 3.3. SOM Application and Statistical Analysis

**Figure 3:**
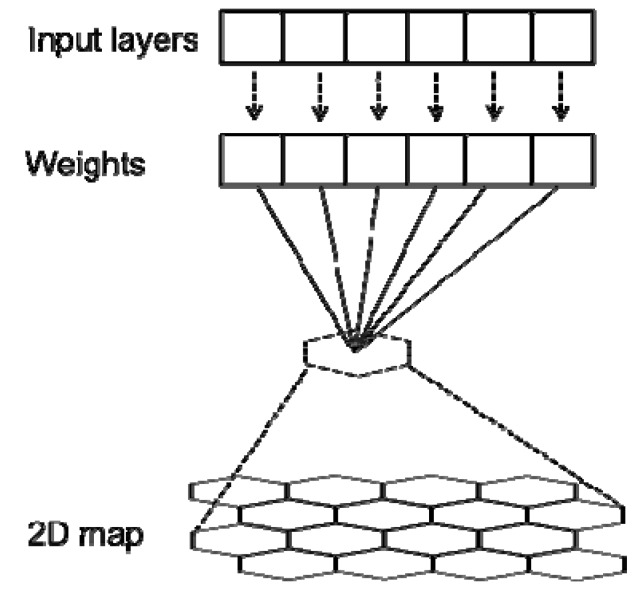
Illustration of how an SOM works.

A SOM algorithm also known as Kohonen Map or Self-Organizing Feature Map, is an unsupervised neural network based on competitive learning [50,51]. It projects high-dimensional input data onto a low dimensional (usually two-dimensional) space. The machine learning is accomplished by first choosing an output neuron that most closely matches the presented input pattern, then determining a neighborhood of excited neurons around the winner, and finally, updating all of the excited neurons [52]. This process iterates and fine tunes, and it is called self-organizing. The outcome weight vectors of the SOM nodes are allocated to have characteristic data patterns. The similar patterns based on k-means are combined with neighboring regions on the map, while dissimilar patterns are located further apart. An illustration of the flow chart of a SOM application is given in Figure 3. Detailed methodological aspects can be found in other computational papers [26,51,53].

In this study, the size of data used in training was 253 cases (*i.e.*, unit towns) multiplied by 20 environmental parameters (Table 2). Map size determination is one of the important features in any SOM application [53]. The optimum map size is selected based on minimum values for quantization error (QE) and topographic error (TE) [54,55]. QE exhibits the average distance between each data vector and its ‘best matching unit’ (BMU), and thus measures map resolution. TE represents the proportion of all data vectors for which 1st and 2nd BMUs are not adjacent, and is used for the measurement of topology preservation [56]. Moreover, QE and TE were adopted to adjust the obtained number of map units, therefore, to minimize errors in performance standard setting. Once the SOM had converged, the U-matrix and K-means algorithm were used in order to find clusters in the nodes of the SOM. To select the best patterning among partitions with different numbers of clusters, the Davies-Bouldin index (DBI) [57] was calculated. The smaller the DBI, the better the clustering. Calculations can be made by using the SOM Toolbox package for Matlab [58]. Cross competitive learning similar patterns are mapped onto neighboring regions on the map, while dissimilar patterns are located further apart (see e.g., [20,57] for further details).

**Figure 4:**
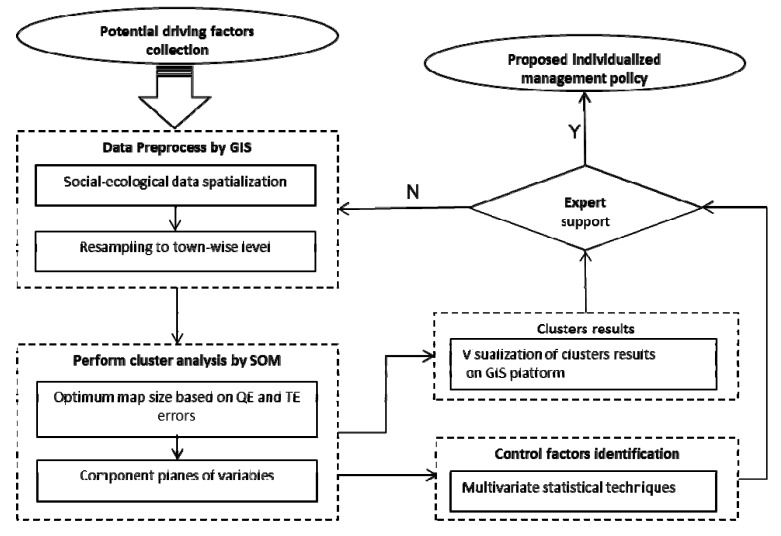
Flow chart of SOM for heavy metals socio-ecological pattern identification.

SOM was used to classify the region. A statistical analysis method was employed to facilitate the understanding of the relationship between heavy metals risk index and social-ecological factors. Correlation coefficients were determined using Spearman’s rank correlation test where p-values less than 0.05 were considered statistically significant [59]. Data were subjected to one-way ANOVA and Duncan’s test was used for multiple comparisons among the zones. All statistical analysis was performed using the SPSS 17 statistical package. Figure 4 depicts the complete framework of the present study.

## 4. Results

### 4.1. SOM Application and Clustering

**Figure 5:**
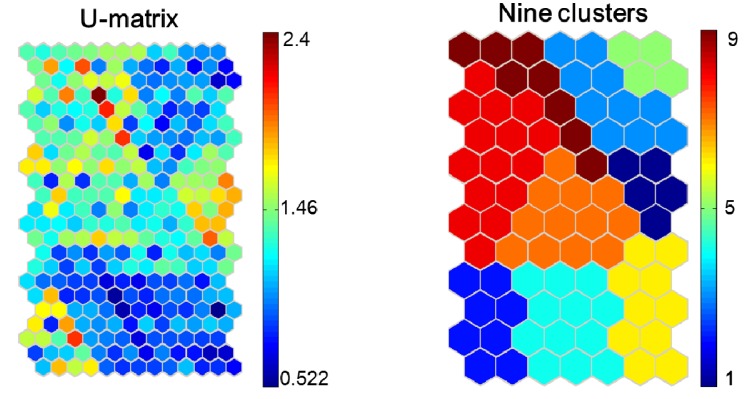
The U matrix and cluster arrangements of nine clusters for the variables (*i.e.*, 253 towns); each node on the U matrix describes the Euclidean distance between nodes in the SOM; therefore there is one node on the U matrix for every adjacent node on the SOM. Red U-matrix node indicates a large distance and blue nodes indicate a small distance.

The QE and TE are summarized at the different map sizes (from 40 to 198 map units) in Table 3. An 84-unit map (12 × 7) was selected with a quantization error of 1.809 and a topographic error of 0.0079, as it exhibited the smallest quantization error and topographic error values among the models. Figure 5 shows the U-matrix and cluster arrangements of nine clusters for the variables using the SOM model. U-matrix is the method for discriminating between the groups (nine clusters), and indicating the distances between the groups. The k-means also shows nine clear clusters based on the minimum DBI (DBI = 0.89) (Table 4). The clusters defined by the U-matrix and k-means methods were consistent with each other. Thus, the communities were classified into nine groups (1–9) based on the U-matrix (Figure 5).

**Table 3:** Map quality measures at different map sizes of the trained SOM.

Map Size	8 × 5 = 40	9 × 6 = 54	10 × 7 = 70	12 × 7 = 84	12 × 8 = 96	13 × 9 = 117	14 × 10 = 140	16 × 11 = 176	18 × 11 = 198
QE	2.727	2.558	2.4254	1.809	2.22	2.1204	1.9893	1.8938	1.8106
TE	0.008	0.012	0.0158	0.0079	0.0119	0.0237	0.0224	0.0119	0.0119

**Table 4:** Davies-Bouldin index (DBI) of k-means clustering at different number of clusters on the trained SOM.

Number of Clusters	2	3	4	5	6	7	8	9	10
DBI	1.32	1.08	1.07	0.91	1.04	1.01	0.93	0.89	0.93

**Figure 6:**
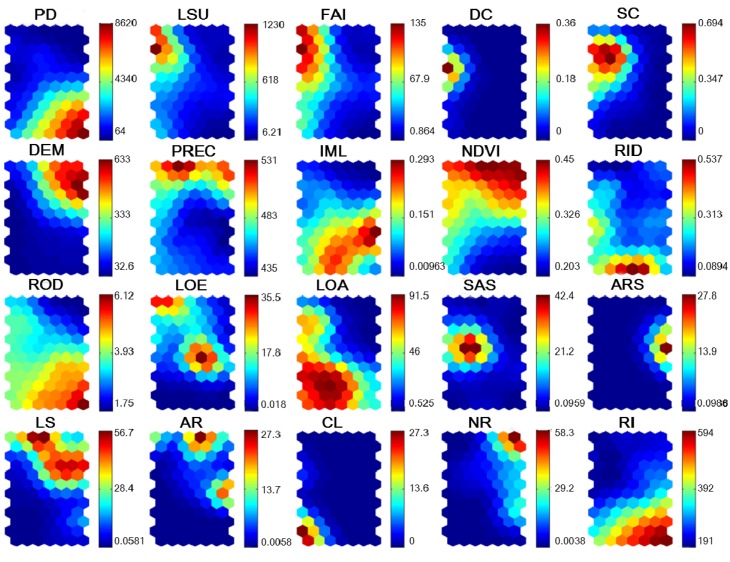
Component maps of the SOM model. The name of each panel represents the input parameters shown as the above. The information of parameter name abbreviation can be found in Table 2.

The distribution patterns of 20 input variables on the SOM plane are shown in Figure 6. The socio-geographical characteristics strongly affected the concentration of heavy metals in soil. The map unit in lower and right nodes showed higher scores for RI. Among these factors, PD, IML and ROD were similarly distributed to the RI. DEM, PREC and NDVI tended to be distributed in the upper left corner, which was relatively opposite to the RI. Agricultural inputs (LSU and FAI) have no significant contribution to the RI, and show higher values in the upper left corner. DC and SC showed a similar distribution, and tended to be related to the agricultural inputs. Soil-forming parent materials might influence the RI. However, this relationship is still unclear in this figure. SOM has the ability to express non-linear relationships, and the complexity in every variable was detected and included in the SOM maps.

### 4.2. Descriptive Cluster Statistics

#### 4.2.1. Pattern of Heavy Metals Contamination

**Figure 7:**
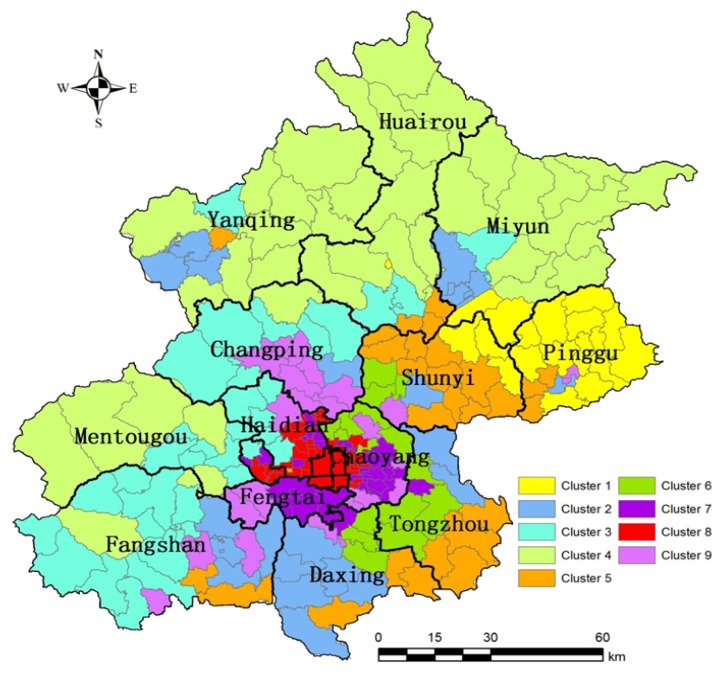
Spatial distribution of the clustering results in Beijing.

When considering the differences in factors among the nine clusters, variance analysis was employed and the results are shown in Table 5. For example, PREC, NDVI, LS were higher in cluster 1, while other land use cover PD, RID and ROD were lower in this pattern. DEM and NDVI were both higher in cluster 1, 3 and 4. Livestock Units (LSU), agricultural land (DC and SC), and soil Soil-forming parent material (CL) were higher in cluster 2, 5, and 6. Among the nine clusters, the demographic driver (PD), critical land use cover (IML and ROD), and state risk (RI) showed relatively high values in cluster 7, 8, and 9. These clusters indicate different interaction patterns between ecological systems for heavy metals contamination. For the spatial distribution of nine clusters on the GIS platform (Figure 7), cluster 1 mainly located in the Pinggu district, including parts of Miyun and Shunyi. 

**Table 5:** The mean value and standard error of environment variables in each cluster defined in SOM.

Variables	Clusters								
	1	2	3	4	5	6	7	8	9
PD	196.4 (230.98) ef	1,254.55 (1,572) de	877.23 (1,889.3) ef	61.42 (69.25) f	576.46 (1,538.48) ef	2,078.62 (2,245.1) cd	5,864.87 (2,199.98) b	7,718.2 (2,483.98) a	2,833.9 (2,736.27) c
LSU	443.87 (686.3) b	363.22 (396.16) bc	100.14 (106.67) d	61.85 (61.52) d	835.39 (843.66) a	351.37 (300.94) bc	98.42 (150.75) d	23.64 (143.4) d	189.92 (220.14) cd
FAI	76.59 (67.79) b	57.74 (44.97) bc	15.4 (25.46) d	7.31 (8.08) d	112.68 (68.54) a	54.83 (39.68) c	11.84 (14.77) d	3.87 (14.58) d	24.96 (24.03) d
DEM	203.84 (151.56) c	113.79 (159.38) d	363.98 (237.79) b	588.52 (213.74) a	51.25 (89.47) d	33.59 (6.99) d	48.91 (23.68) d	57.05 (17.93) d	57.63 (27.33) d
PREC	533.39 (37.7) a	452.41 (28.07) de	452.58 (24.73) de	502.39 (31.96) b	474.61 (28.58) d	463.11 (13.74) cd	451.76 (15.28) de	442.93 (10.97) e	440.38 (31.29) e
IML	0.03 (0.02) d	0.12 (0.08) bc	0.09 (0.07) c	0.01 (0.01) d	0.08 (0.05) c	0.14 (0.04) b	0.23 (0.1) a	0.16 (0.12) b	0.21 (0.09) a
NDVI	0.43 (0.04) a	0.31 (0.04) d	0.38 (0.04) b	0.44 (0.04) a	0.36 (0.04) c	0.29 (0.04) e	0.22 (0.04) g	0.21 (0.03) g	0.27 (0.04) f
RID	0.07 (0.08) e	0.21 (0.2) bcd	0.15 (0.1) de	0.17 (0.08) cd	0.27 (0.14) bc	0.3 (0.14) b	0.48 (0.3) a	0.21 (0.18) bcd	0.19 (0.16) cd
ROD	2.72 (0.73) h	3.39 (0.75) ef	2.94 (1.22) fg	1.82 (0.45) h	3.76 (0.44) de	4.08 (0.69) cd	4.93 (1.46) ab	5.53 (1.58) a	4.42 (1.5) bc
DC	0 (0) b	0.01 (0.02) b	0 (0.01) b	0 (0) b	0.22 (0.26) a	0.02 (0.05) b	0 (0) b	0 (0) b	0 (0) b
SC	0.04 (0.05) c	0.37 (0.36) b	0.03 (0.08) c	0.01 (0.03) c	0.62 (0.31) a	0.05 (0.07) c	0 (0) c	0 (0) c	0.04 (0.16) c
LOE	26.65 (21.14) a	10.57 (15.59) bc	7.71 (14.29) bcd	5.18 (11.79) bcd	12.77 (24.65) b	0 (0) d	0.15 (0.83) d	0.78 (4.61) cd	22.15 (31.57) a
LOA	8.26 (17.15) e	34.77 (32.39) d	3.1 (8.14) e	1.41 (3.43) e	67.39 (24.16) bc	76.83 (13.94) ab	85.63 (23.04) a	35.57 (31.03) d	58.53 (41.87) c
SAS	0.91 (3.87) c	41.05 (31.32) a	1.05 (3.74) c	1.01 (4.94) c	12.17 (14.88) b	0.5 (1.99) c	1.92 (8.52) c	1.29 (6.15) c	2.27 (5.4) c
ARS	0 (0) b	0.1 (0.5) b	13.38 (18.36) a	0.12 (0.7) b	0 (0) b	0 (0) b	0 (0) b	0.06 (0.33) b	1.41 (6.78) b
LS	56.52 (32.27) a	6.54 (19.03) c	49.39 (30.89) a	22.95 (24.1) b	3.55 (7.49) c	0.07 (0.23) c	2.28 (6.47) c	7.43 (20.67) c	2.14 (4.92) c
AR	7.61 (24.63) b	2.2 (6.06) b	6.58 (13.99) b	15.76 (20.48) a	0.03 (0.15) b	0 (0) b	0 (0) b	0 (0) b	7.13 (23.67) b
CL	0 (0) b	0.08 (0.27) b	0 (0) b	0 (0) b	2.36 (7.08) b	22.6 (13.45) a	0.34 (1.87) b	0 (0) b	0.04 (0.18) b
NR	0.05 (0.22) c	3.13 (9.04) c	15.09 (17.91) b	41.01 (30.12) a	1.18 (5.79) c	0 (0) c	0.12 (0.64) c	8.81 (22.16) bc	2.17 (9.32) c
RI	208.47 (40.76) e	203.35 (31.36) e	274.79 (72.14) d	221.71 (37.2) e	198.81 (73.73) e	365.5 (118.24) b	513.48 (88.93) a	553.65 (78.3) a	321 (124.45) c

Note: Different alphabet letters indicate significant differences among the clusters based on Dunn’s multiple comparison tests at the 0.05 levels.

A dozen regional patches were found in cluster 2 in the city’s peripheral suburbs. Cluster 3 and cluster 4 locate in the north and west quadrant of Beijing, respectively. Cluster 5 and cluster 6 were found in the vicinity of agricultural land in the east quadrant. Cluster 7, 8 and 9 were toward the city center area, relatively, where high eco-risk areas were observed.

**Table 6:** Concentrations and multiple comparisons of heavy metals of different clusters (milligrams per kilogram).

Element		1	2	3	4	5	6	7	8 *	9
	n	96	189	109	147	287	81	14	-	95
Ni	Means ± SD	29.91 ± 8.65	25.98 ± 8.72	29.53 ± 7.3	31.99 ± 11.87	27.77 ± 9.53	26.65 ± 7.86	32.2 ± 25.14	-	28.65 ± 20.08
	Multiple comparisons	ab	b	ab	a	ab	b	a	-	ab
Cr	Means ± SD	58.07 ± 17.39	53.76 ± 11.85	62.54 ± 13.41	78.11 ± 37.77	57.33 ± 14.14	61.29 ± 14.15	56.61 ± 11.75	-	58.9 ± 10.61
	Multiple comparisons	bc	c	b	a	bc	bc	bc	-	bc
Cu	Means ± SD	53.81 ± 37.18	53.59 ± 35.62	67.51 ± 65.25	62.69 ± 44.47	53.98 ± 40.56	67.68 ± 43.94	79.08 ± 58.14	-	71.44 ± 54.82
	Multiple comparisons	b	b	ab	ab	b	ab	a	-	ab
Zn	Means ± SD	74.23 ± 20.31	70.24 ± 19.57	79.72 ± 23.15	83.61 ± 18.05	72.18 ± 17.55	80.96 ± 28.28	87.51 ± 22.84	-	81.64 ± 22.2
	Multiple comparisons	bcd	d	abc	a	cd	ab	a	-	ab
As	Means ± SD	17.1 ± 10.71	15.47 ± 7.88	18.63 ± 10.75	14.79 ± 7.87	15.17 ± 7.45	16.87 ± 7.61	15.14 ± 6.09-		17.56 ± 7.98
	Multiple comparisons	ab	ab	a	b	b	ab	b	-	ab
Cd	Means ± SD	0.24 ± 0.15	0.24 ± 0.15	0.24 ± 0.19	0.22 ± 0.12	0.23 ± 0.29	0.25 ± 0.12	0.33 ± 0.29	-	0.25 ± 0.2
	Multiple comparisons	b	b	b	b	b	ab	a	-	ab
Pb	Means ± SD	49.93 ± 48.26	44.25 ± 34.48	53.98 ± 39.64	55.58 ± 61.36	43.76 ± 30.07	54.65 ± 34.69	65.44 ± 45.49	-	63.08 ± 43.5
	Multiple comparisons	ab	b	ab	ab	b	ab	a	-	a
Hg	Means ± SD	0.15 ± 0.28	0.13 ± 0.1	0.28 ± 0.48	0.2 ± 0.24	0.14 ± 0.15	0.29 ± 0.19	0.94 ± 0.73	-	0.42 ± 0.55
	Multiple comparisons	d	d	c	cd	d	c	a	-	b

Notes: * There was no soil sample located in Cluster 8; Different alphabet letters indicate significant differences among the clusters based on Dunn’s multiple comparison tests at the 0.05 levels.

#### 4.2.2. Differences in Heavy Metals Contamination among SOM Clusters

Table 6 illustrates the soil samples variations in the amounts of Ni, Cr, Cu, Zn, As, Cd, Pb, and Hg in Beijing under different clusters. The ANOVA results indicated that most of clusters had significant differences in the accumulation of heavy metals in Beijing. Most of the elements had the highest contents in cluster 7, except for Cr and As. Simultaneously, relatively high contents of all elements were found in cluster 9. Cluster 7 and cluster 9 are located in zone III, where there is mainly urban land. Ni, Cr, and Zn were relatively higher in cluster 4, which consisted of the communities located in higher DEM, precipitation and Normalized Difference Vegetation Index (NDVI). The distribution of As was homogeneous in each cluster, which might indicate that the source of As is from the natural indigenous soil minerals in Beijing. The contents of Cu and Zn are not greater in cluster 2, 5, and 6 (zone II) than the cluster 7 and 9 (zone III), suggesting that fertilizers and agricultural input had less impact than the ritual casting of bronze which started over 4,000 years ago and automobile tire wear in modern Beijing. For Cd, Pb and Hg, the classification results provided the best outcomes. The distribution of these elements was separated into two dimensions: a higher concentration in clusters 7 and 9 (urbanized areas), while a lower concentration in the others.

### 4.3. Relationships between Soil Heavy Metals Risk and Environmental Variables

Correlation analysis was performed to investigate the relationships between environmental variables and soil heavy metals among the nine clusters. Most comparison cases in the Spearman correlation matrix showed a clear statistical significance when community characterizations were conducted (Table 7). 

**Table 7:** Spearman rank correlation coefficient of variables and heavy metal RI to the town-wise data (n = 253) of nine clusters.

	RI_1	RI_2	RI_3	RI_4	RI_5	RI_6	RI_7	RI_8	RI_9
PD	0.05	−0.092	0.409 *	−0.031	0.925 **	0.812 **	0.313	0.268	0.05
LSU	0.495 **	−0.371	−0.203	0.248	0.707 **	−0.141	−0.183	−0.214	0.495 **
FAI	0.312	−0.276	0.459 **	0.358 *	0.792 **	−0.564 *	−0.203	−0.277	0.312
DEM	−0.284	0.022	−0.262	−0.217	0.055	0.611 *	−0.301	−0.569 **	−0.284
PREC	0.088	−0.237	−0.289	0.534 **	−0.385 *	−0.511 *	0.337	0.783 **	0.088
IML	0.369 *	0.201	0.254	−0.008	0.646 **	0.605 *	−0.272	−0.738 **	0.369
NDVI	−0.155	−0.101	−0.262	−0.158	−0.709 **	−0.828 **	−0.398 *	−0.560 **	−0.155
RID	0.01	0.205	0.122	0.057	−0.449 *	0.311	0.237	0.284	0.01
ROD	−0.14	−0.095	0.648 **	−0.09	0.091	0.245	0.352	0.423 **	−0.14
DC	−0.288	−0.111	−0.055	0.262	0.011	−0.488	−0.15	−0.306	−0.288
SC	−0.205	−0.184	−0.258	−0.218	−0.484 **	−0.586 *	−0.495 **	−0.442 **	−0.205
LOE	−0.017	0.021	−0.434 **	−0.333 *	0.032	**a**	0.062	−0.17	−0.017
LOA	0.145	0.344	**a**	−0.135	0.026	0.072	0.039	−0.109	0.145
SAS	0.138	−0.256	0.13	−0.268	−0.173	−0.255	0.167	−0.141	0.138
ARS	0.438 **	0.274	0.406 *	0.207	**a**	**a**	**a**	−0.185	0.438 *
LS	0.478 **	0.097	−0.112	0.166	−0.127	−0.167	0.192	−0.355 *	0.478 *
AR	−0.194	−0.236	−0.269	−0.420 **	−0.127	**a**	**a**	**a**	−0.194
CL	−0.132	0.39	**a**	**a**	0.026	−0.034	0.07	**a**	−0.132
NR	0.212	−0.531 **	0.15	0.24	−0.13	**a**	0.062	−0.373 *	0.212

Notes: *. Correlation is significant at the 0.05 level (2-tailed); **. Correlation is significant at the 0.01 level (2-tailed); a. Cannot be computed because at least one of the variables is constant.

Different relationships were detected among the nine clusters (*i.e.*, NDVI is strongly negatively related to the soil heavy metal risk in clusters 5, 6 and 8, while has no significant correlation coefficients in the others). Many more factors have significantly correlated coefficients (*p* < 0.01) in clusters 5, 6, and 8, indicating that the function of soil heavy metals risk is complicated in these clusters. In contrast, the structure and function are relatively simple in clusters 2, 7, and 9. 

As for concrete environmental variables, the increased population density also caused soil heavy metals risk in clusters 3, 5 and 6 located at the suburban vicinity. Livestock and IML were positively related with RI in cluster 1 and cluster 5. Among the factors, most of them were related to the risk of heavy metals in Beijing. However, the area of double cropping, loam and clay in communities were not significantly correlated with RI. 

## 5. Discussion

Ecology is not able to quantify all the relations between exposure and effects of contaminants such as heavy metals, because of the complexity of the sources and their interactions with human well-being. Thus, ecologists and environmental managers endeavor to find environmental indicators which can represent parts of these complex environmental relationships. In recent decades, there have been a series of classifications of different subjects, such as aquatic ecosystem classifications (AEC), biodiversity conservation classification and forest service classification. Social-ecological patterns represent the comprehensive interaction of substance and energy, which form the live organisms and the environment. Thus, difference-oriented policies should be made based on the local ecosystems, which share a number of basic structural and functional characteristics [10,13]. In this study, SOM was performed to classify the region of Beijing into nine clusters at the town-wise level, and a number of the major factors that control heavy metals contamination were identified in each cluster. 

SOM have been proven to be a promising tool for describing the evolution of metal accumulations in terrestrial ecosystems. The SOM projection shifts the complicated structure from high dimensional arrays into the lower dimensional clusters based on the neighborhood relations, which is important to ecological classifications for environmental management. For instance, the spatial generalization of environmental data are measured at single sites as well as in dynamic global change modeling in land use or environmental planning [11,60,61]. Land classifications provide spatial reference systems that may indicate long-term effects (responses), for example, structural and functional, resulting from the bioaccumulation of contaminants. Such effects depend both on the stress intensity and on the ecological characteristics and related sensitivities of the land unit which is exposed [10]. 

From the results of this study, it can be concluded that the heavy metals contamination patterns are strongly related to human populations, IML, and ROD distribution. Additionally, vegetation and geographical aspects of the land also affected heavy metals concentration. We could hypothesize the sequential pattern as the following: (1) high heavy metals risk areas locate in urbanized areas that exhibit high population and low elevation, which are suitable for increased road and IML density; (2) these areas, are more frequently invaded by vehicular traffic volumes and urbanization; (3) therefore, land use management on community units (at a town level) is needed in the more populated areas in order to reduce the heavy metals risk.

Results from the ANOVA analysis of the element contents show differences among the clusters indicating that heavy element contents in clusters 7, 8 and 9 (the urban area) were higher than the other clusters. Cu, Zn, Cd, Pb and Hg in urban soils usually come from gasoline, car components, oil lubricants, and industrial and incinerator emissions [6,34,62,63,64]. Although leaded gasoline has been banned in Beijing since 1997, the impact on this area may last for the coming years [6], and also decoration waste deposition from the repairs of time-honored parks was considered [29]. The source of Cd in this area may be from coal combustion, solid wastes such as plastics and automobile tires [29,34]. The casting of bronze ritual figures and the automobile tire wear were considered to be the main sources of Cu and Zn. Hg mainly came from the atmospheric deposition along the roadside. Therefore, traffic volume regulations and air cleaning plans should be implemented in these areas. Clusters 2, 5 and 6 are mainly located in the agricultural areas and are far away from the central city, while Cr, Cu, Zn, Hg exhibited ascending trends among them. These may illustrate that the sources were both from industrial emissions and agrochemical inputs [8,31,33]. Therefore, green agriculture is suitable in these communities, which should decrease the use of the Cd-containing, Cu-containing agricultural material inputs from agricultural activities. Heavy metals in clusters 1, 3 and 4 showed higher level elevation, vegetation index, precipitation and parent materials diversity than other clusters. Restoration management was needed in these areas, which was consistent with the report of the Beijing Government [65]. 

**Figure 8:**
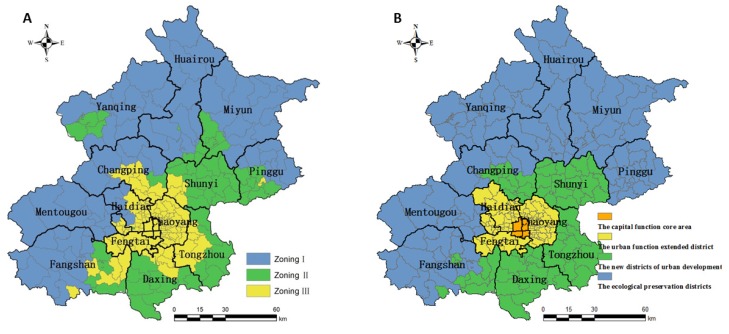
(**A**) Further presentation of clustering results and (**B**) Major function oriented zone of Beijing.

To meet the land-use management needs, besides the identification of metal accumulation spatial patterns, the consecutive spatial patterns are also an important component of zoning [66]. Integrating the fragmentary zones benefits soil trace elements management at the generalized scale. According to the analysis in Section 4.2, in the present study, the main soil heavy metals contamination patterns could be drawn in three zones. Figure 8A illustrates the spatial distribution of communities in Beijing in accordance with clustering results analysis and SOM model. 

Zone I was located in the mostly mountainous areas and had relatively high vegetation cover in the west and northwest of Beijing (Figure 2A,B). Zone II consisted of the communities located in the peripheral suburbs, whose predominant land use type was agricultural land (Figure 2B). Furthermore, Yanqing was also detected and grouped into zone II, where was used to be a large state farm. Zone III Ⅲ was formed with the remaining area, and located inside the 6th ring, and this contains the most recently urbanized area of Beijing (Figure 2B–D). Regarding the clusters, clusters 1, 3 and 4 were mainly contained in zone I, clusters 2, 5, and 6 were attached to zone II, and the remainder were located in zone III. In order to prove the cluster results we proposed, the current development plan of Beijing was employed for analysis. 

In China, the concept of major function oriented zone (MOFZ) was proposed to achieve coordinated regional development and environmental protection based on the territorial functions [67,68]. More recently, the MOFZ of Beijing municipality was released [69] and it clearly stated four function-oriented zones (the capital function core area, the urban function extended district, the new districts of urban development and the ecological preservation districts, see Figure 8B). However, the four districts may not be satisfactory for concrete environmental management policy formulations. The nine zones proposed in the present study were clustered by a bottom-up approach based on SOM. Although the MOFZ was made by political or legal means, the integrated zones in accordance with the SOM and multivariate statistical techniques were consistent with the MOFZ of Beijing, indicating that the classification in this literature was reasonable and suited for the actual conditions of the study area. However, a slight difference could be found between Figure 8A,B. The former zones were not limited by the administrative boundary lines. For example, the propagation pathway of zone III was similar to the new districts of urban development in the downwind southeast quadrant of the city. Moreover, the direction of expansion of the Changping and Fangshan districts should be considered by government decision makers.

## 6. Conclusions

Ecoregional synthesis and management classification are the key issues of agricultural soil heavy metal environmental monitoring and management, linking the metals accumulation at individual soil sites with the social-ecological dataset of the area managed [10,13,14]. A total of 1,018 soil samples were investigated in order to conduct a comprehensive monitoring and management of the soil heavy metals in Beijing. Social-ecological datasets were developed according to the possible potential sources of the heavy metals. Self-organizing map (SOM) and multivariate statistics were employed to cluster the data into nine habit types which could reveal the homogeneity and regularity of heavy metal migration. It can be used to further soil environment management and land use planning at a province level.

The main outcomes of the study can be drawn as follows: (i) SOM was verified to be a promising approach for pattern recognition and, in particular, for delineating social-ecological patterns of soil heavy metals; (ii) the main factors that influence the heavy metal concentration in Beijing were associated with the population density, vegetation index, industrial and mining land percent and road density—this is useful information for reference in future research; (iii) the social-ecological patterns of heavy metals in Beijing were detected in nine clusters and mainly categorized into three zones. The results are critical for improving the efficiency of the soil heavy metals management; (iv) classification of social-ecological patterns on soil heavy metals (SEPHM) provides a great deal of information enhancing the risk status source identification at the community scale, although the temporal spatial patterns were merely considered in this study. The spatialization of other factors such as the atmospheric deposition, sewage irrigation and sewage sludge may modify the patterns in a greater refinement. Designing a theoretical framework for combining these perspectives will be an exciting open problem for future analysis.
